# The complete mitochondrial genome of comma, *polygonia c-aureum* (Lepidoptera: Nymphalidae: Nymphalinae)

**DOI:** 10.1080/23802359.2017.1419091

**Published:** 2017-12-24

**Authors:** Qing-Hui Shi, Jian-Hong Xing, Xi-Hua Liu, Jia-Sheng Hao

**Affiliations:** a Fujian Provincial Key Laboratory of Resources and Environment Monitoring & Sustainable Management and Utilization, Sanming University, Sanming, PR China;; b Laboratory of Molecular Evolution and Biodiversity, College of Life Sciences, Anhui Normal University, Wuhu, PR China

**Keywords:** Mitochondrial genome, Nymphalidae, Nymphalinae, *Polygonia c-aureum*

## Abstract

The mitochondrial genome (mitogenome) of *Polygonia c-aureum* (Lepidoptera: Nymphalidae: Nymphalinae) is determined to be 15,209 bp in length and shows AT bias (80.6%). Similar to other butterflies, it contains 37 typical mitochondrial genes and one AT-rich region (D-loop). All protein-coding genes (PCGs) started with ATN, except for *cox1* gene with CGA(R), which is often found in other butterflies, and seven PCGs harbour the typical stop codon TAA, whereas *cox1*, *cox2*, *nad3*, *nad5*, *nad4* and *nad1* end with a single T. The *rrnL* and *rrnS* genes are 1332 bp and 773 bp in length, respectively. The 342 bp AT-rich region contains non-repetitive sequences, but harbour several features common to the lepidopterans, including the motif ATAGA followed by a 19-bp poly-T stretch and a microsatellite-like (TA)_8_ element preceded by the ATTTA motif. The complete mitogenome sequence provided here would be useful for further understanding the taxonomy and phylogeny of Nymphalinae.

The subfamily Nymphalinae (Lepidoptera: Nymphalidae) comprises about 500 species and is distributed nearly all around the world (Harvey 1991). However, the taxonomy and phylogeny of Nymphalinae are still standing as a controversial issue, and need to be elucidated (Wahlberg et al. 2009; Shi et al. 2015). In recent decades, the insect mitochondrial genomes (mitogenomes) have been widely used as an informative molecular marker for phylogenetic and population genetic studies at various hierarchical levels, due to their unique features (Boore 1999; Salvato et al. 2008; Wu et al. 2014; Timmermans et al. 2016).

Here, we sequenced and characterized the complete mitogenome of *Polygonia c-aureum* (tribe Nymphalini). Adult individuals of *P. c-aureum* were netted at Nanjing, Jiangsu province, China (coordinates: E118^°^46’, N32^°^03’) on July 2012. After morphological identification, fresh individuals were preserved in 100% ethanol and kept in the laboratory at −20 °C under the accession number SQH-20120718. Total genomic DNA was extracted from the thorax muscle of a single individual using the Sangon Animal genome DNA Extraction Kit (Shanghai, China). The resultant reads were assembled and annotated using the BioEdit 7.0 (Hall 1999) and MEGA6 software (Tamura et al. 2013) with reference to mitogenome of *Hypolimnas bolina* (GenBank accession NC_026072) (Shi et al. 2015).

The complete mitogenome of *P. c-aureum* is a circular molecule of 15,209 bp in size (GenBank accession MF407452), containing typical insect 13 protein-coding genes (PCGs), two ribosomal RNA genes (rRNAs) and 22 transfer RNA genes (tRNAs), along with an AT-rich control region. Its gene arrangement and orientation are identical to those of other known nymphalid mitogenomes (e.g. Wu et al. 2014; Shi et al. 2015; McCullagh and Marcus 2015; Timmermans et al. 2016). The A + T content of the *P. c-aureum* mitogenome is 80.6%, which is generally in accordance with other nymphalid mitogenomes. All PCGs are initiated by typical ATN codons, except for *cox1* gene, which is started with the unusual CGA(R) as observed in most of the other sequenced nymphalid butterflies (Kim et al. 2010; Hao et al. 2013; Gan et al. 2014; Shi et al. 2015). Seven PCGs have a complete stop codon (TAA), while *cox1*, *cox2*, *nad3*, *nad5*, *nad4* and *nad1* end with a single T. All tRNAs harbour the typical predicted secondary cloverleaf structures except for the *trnS1*, as seen in all other determined butterflies (Hao et al. 2013; Shi et al. 2015). The length of *rrnL* and *rrns* genes is 1332 bp and 773 bp, respectively. The putative AT-rich region is 342 bp long (93.8% A + T content) with several structures characteristic of lepidopterans.

We performed a maximum-likelihood (ML) phylogenetic analysis with RAxML (version 7.2.6) using nucleotides sequences of 13 PCGs and two rRNAs to understand the phylogenetic relationship of *P. c-aureum* with other nymphalids (see Figure 1 for details). ML analysis exhibited that *P. c-aureum* formed a monophyletic group with other Nymphalinae species, which were recovered as the sister group to a clade containing the nymphalid subfamilies Apaturinae and Biblidinae with strong support value.

**Figure 1.**
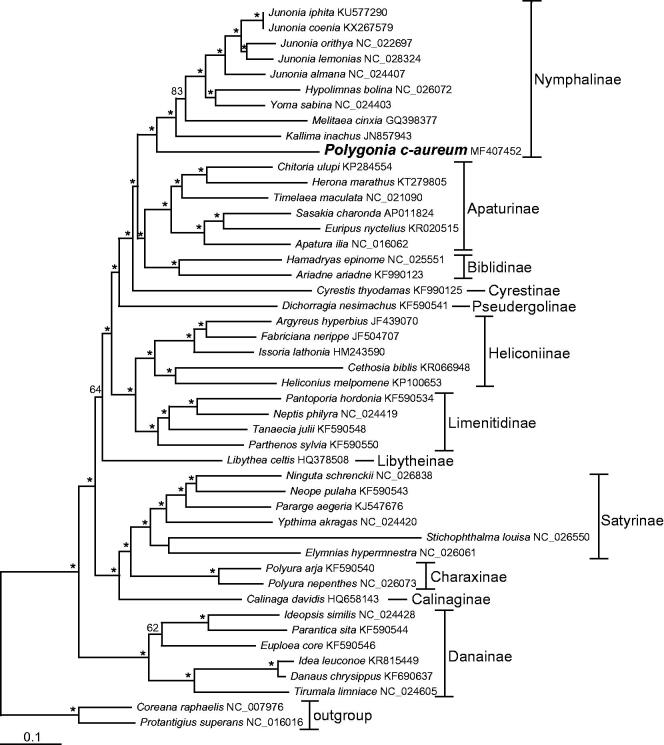
The maximum-likelihood (ML) phylogenetic tree of *Polygonia c-aureum* and other nymphalid species. Phylogenetic reconstruction was done from a concatenated matrix of 13 protein-coding mitochondrial genes and two ribosomal RNA genes regions in the mitochondrial genome. The numbers beside the nodes are percentages of 1000 bootstrap values (* ≥85%). Alphanumeric terms indicate the GenBank accession numbers.
